# Pharmacologic management of postoperative sleep disturbance in breast cancer

**DOI:** 10.3389/fphar.2026.1844297

**Published:** 2026-07-29

**Authors:** Fan Bu, Shulin Zeng, Qinzhe Liu, Zhengchi Lou, Chao Ma, Yueming Peng, Lile Xiong, Yi Wen, Lan Qin

**Affiliations:** 1 Department of Neurology, Dongzhimen Hospital Affiliated to Beijing University of Chinese Medicine, Beijing, China; 2 Department of General Surgery, Qidong Hospital of Traditional Chinese Medicine, Qidong, Jiangsu, China; 3 Department of Orthopedics, Dongzhimen Hospital, Beijing University of Chinese Medicine, Beijing, China; 4 Department of Traditional Chinese Medicine, The Third Affiliated Hospital of Henan Medical University, Xinxiang, Henan, China; 5 Department of Oncology, Pingyang Hospital of Traditional Chinese Medicine, Zhejiang, China; 6 Department of Nursing, Shenzhen People’s Hospital (The First Affiliated Hospital, Southern University of Science and Technology, The Second Clinical Medical College, Jinan University), Shenzhen, Guangdong, China; 7 Department of Thoracic Surgery, Shenzhen People’s Hospital (The First Affiliated Hospital, Southern University of Science and Technology, The Second Clinical Medical College, Jinan University), Shenzhen, Guangdong, China; 8 Department of Neonatology, Shenzhen People’s Hospital (The First Affiliated Hospital, Southern University of Science and Technology, The Second Clinical Medical College, Jinan University), Shenzhen, Guangdong, China

**Keywords:** breast cancer surgery, melatonin, orexin receptor antagonists, patient safety, pharmacologic management, postoperative sleep disturbance

## Abstract

Postoperative sleep disturbance (PSD) is a common yet underrecognized complication after breast cancer surgery and is associated with pain, fatigue, affective symptoms, impaired quality of life, and delayed postoperative recovery. Its management remains challenging because symptom presentations are clinically heterogeneous, and treatment decisions must balance therapeutic efficacy with safety within the broader context of breast cancer care and survivorship. This narrative review summarizes the current evidence on PSD after breast cancer surgery, with particular emphasis on pharmacologic management, therapeutic positioning, and breast cancer-specific safety considerations. Available evidence suggests that a phenotype-guided pharmacologic management framework may be clinically useful, although this approach remains conceptual and has not yet been prospectively validated in postoperative breast cancer populations. Short-course hypnotics may be considered for acute insomnia characterized by prominent nocturnal hyperarousal, whereas melatonin and melatonin receptor agonists may be particularly relevant when circadian disruption predominates. In contrast, gabapentinoids and multimodal analgesic strategies may be more appropriate for pain-dominant or mixed symptom presentations. Across drug classes, treatment selection should account for cumulative sedative burden, respiratory risk associated with concurrent opioid use, next-day cognitive adverse effects, and endocrine therapy context, including avoidance of strong CYP2D6 inhibitors in patients receiving tamoxifen. Among non-pharmacologic interventions, cognitive behavioral therapy for insomnia (CBT-I) is best positioned as the leading adjunctive strategy, particularly for persistent symptoms or during medication de-escalation. Bright light therapy, exercise-based rehabilitation, mindfulness-based interventions, and acupuncture may provide selective supportive benefits in appropriately selected patients, although their roles are generally complementary rather than primary. Overall, PSD after breast cancer surgery should be recognized as a clinically meaningful component of postoperative recovery and survivorship care. Future studies should prioritize standardized sleep outcomes, objective monitoring where feasible, clearer phenotype definition, and integrated efficacy–safety assessment to support more individualized and mechanism-informed management.

## 1 Introduction

Breast cancer remains the most frequently diagnosed malignancy in women worldwide, and continued improvements in survival have increased the importance of symptom control, functional recovery, and quality of life during the perioperative and survivorship phases. Among the symptoms that affect postoperative recovery, sleep disturbance is common yet often underrecognized after breast cancer surgery. In this setting, poor sleep is associated with pain, fatigue, anxiety, depressive symptoms, impaired daytime functioning, and delayed recovery, and it may contribute to a broader symptom cluster that further compromises wellbeing during early survivorship (Adalbe et al., 2024).

Postoperative sleep disturbance (PSD) after breast cancer surgery is clinically heterogeneous. In some patients, insomnia is characterized predominantly by nocturnal hyperarousal and difficulty initiating sleep, whereas in others, sleep disruption appears to be more closely linked to postoperative pain, circadian misalignment, medication effects, emotional distress, or mixed symptom presentations (Salman et al., 2019; Salman et al., 2021). This heterogeneity has important implications for treatment selection, because optimal management depends not only on the presence of sleep symptoms, but also on the underlying clinical phenotype and the broader perioperative context in which sleep disruption occurs.

Pharmacologic treatment remains a common clinical strategy for acute or clinically significant postoperative insomnia, particularly when symptoms are functionally impairing or closely intertwined with pain and distress. However, pharmacologic management in women with breast cancer requires particular caution. The potential benefits of hypnotics, melatonergic agents, gabapentinoids, and related agents must be weighed against cumulative sedative burden, respiratory risk with concurrent opioid use, cognitive adverse effects, and breast cancer-specific considerations such as endocrine therapy context and clinically relevant drug-drug interactions. At the same time, behavioral and circadian-oriented interventions may enhance durability of benefit and support de-escalation of sedative medications when appropriate.

In this narrative review, we summarize current evidence on PSD after breast cancer surgery, with a particular emphasis on pharmacologic management, therapeutic positioning, safety and tolerability considerations, and clinically relevant future directions. Unlike previous reviews that have mainly addressed cancer-related insomnia across heterogeneous tumor types or broad survivorship settings, this review focuses specifically on the postoperative breast cancer context, in which sleep disturbance is shaped by surgical stress, postoperative pain, anesthesia exposure, psychological distress, endocrine symptoms, and adjuvant treatment-related adverse effects. The novelty of this review lies in its breast cancer surgery-specific focus, its integration of breast cancer-related pharmacologic considerations, and its proposal of a phenotype-informed framework for individualized and safety-conscious management. We also discuss the adjunctive role of non-pharmacologic and integrative approaches within a broader breast cancer care framework, with the aim of supporting more individualized, mechanism-informed, and safety-conscious management of sleep disturbance in this population.

## 2 Literature search strategy

This narrative review focuses on the mechanistic, pharmacologic, and translational therapeutic aspects of PSD after breast cancer surgery. To improve transparency, a structured literature search was conducted in PubMed, Web of Science, and Google Scholar through March 2026 using terms related to breast cancer surgery, sleep disturbance or insomnia, and relevant pharmacologic or mechanistic domains, including hypnotics, melatoninergic agents, orexin receptor antagonists, gabapentinoids, multimodal analgesia, circadian disruption, and biomarker-informed approaches. Priority was given to randomized controlled trials, prospective or controlled clinical studies, systematic reviews and meta-analyses, biomarker-informed mechanistic studies in breast cancer populations, and directly relevant preclinical studies. Throughout this review, direct therapeutic evidence is distinguished from indirect clinical extrapolation and from preclinical or hypothesis-generating findings.

As a narrative review, this article aims to provide a clinically oriented synthesis rather than a formal systematic evidence appraisal.

## 3 Epidemiology and clinical characteristics of PSD in breast cancer

Sleep disturbance is among the most prevalent and persistent symptoms across the breast cancer continuum and is particularly prominent during the perioperative and early survivorship phases. A meta-analysis of 51 studies (N = 28,036) reported a pooled prevalence of poor sleep quality of 62% (95% CI: 59–66) among breast cancer survivors (Wen et al., 2023). Sleep problems often emerge during treatment, may worsen around surgery and adjuvant therapy, and can persist well beyond the acute postoperative period (Mohammed, 2021).

In clinical practice, PSD frequently co-occurs with fatigue, anxiety, depressive symptoms, and pain, thereby worsening quality of life and delaying functional recovery (Shamini et al., 2015). Clinical presentations may be predominantly insomnia-related, circadian-disrupted, pain-associated, medication-related, or mixed, underscoring the heterogeneity of sleep complaints in this population. The Pittsburgh Sleep Quality Index (PSQI) and Insomnia Severity Index (ISI) remain the most widely used subjective assessment tools in breast cancer follow-up, whereas objective measures may identify reduced sleep efficiency, increased wake after sleep onset, and broader disruption of sleep continuity (Mesut, 2020). This heterogeneity has important therapeutic implications, because symptom phenotype may influence whether pharmacologic treatment, behavioral intervention, circadian-oriented therapy, or multimodal symptom-cluster management should be prioritized.

## 4 Pathophysiological mechanisms of PSD in breast cancer

**FIGURE 1:**
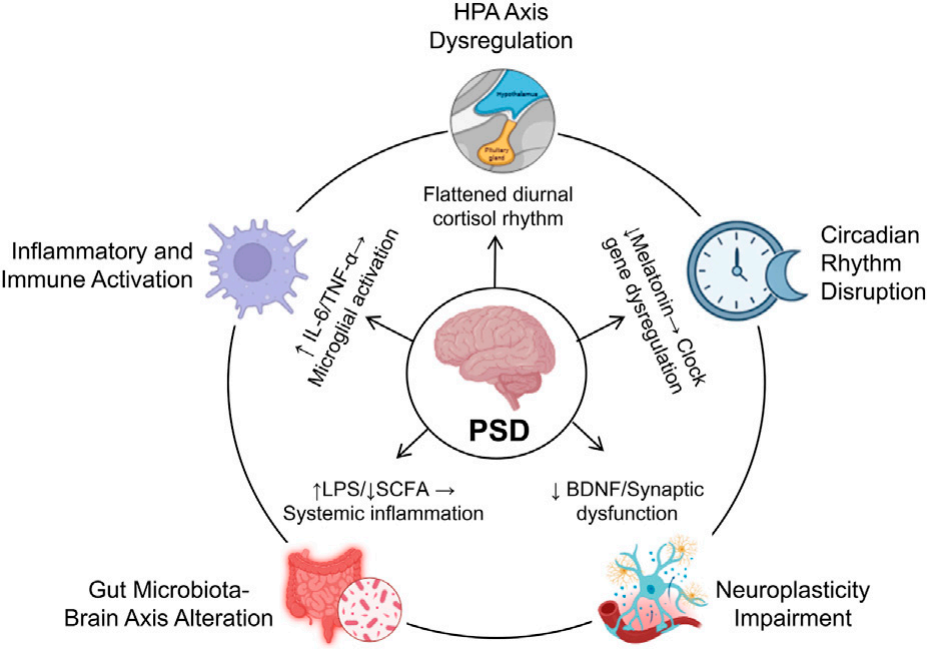
Mechanistic pathways underlying postoperative sleep disturbance (PSD) after breast cancer surgery. This conceptual schematic summarizes the major biological pathways contributing to postoperative sleep disturbance after breast cancer surgery. Surgical tissue injury and nociceptive input initiate inflammatory activation, sympathetic arousal, and hypothalamic–pituitary–adrenal (HPA) axis dysregulation, leading to increased release of proinflammatory mediators and disruption of neuroendocrine homeostasis. These processes interact with circadian and melatonergic disruption, autonomic imbalance, gut–microbiota–brain signaling, and neural plasticity remodeling. Together, these interconnected pathways contribute to sleep fragmentation, nocturnal hyperarousal, affective symptoms, and impaired postoperative recovery. Bidirectional arrows indicate reciprocal interactions among mechanistic domains. Notes:This figure is a conceptual schematic synthesized from published evidence and does not represent original experimental data. Curved arrows indicate bidirectional interactions. Upward and downward arrows (↑/↓) denote relative increases or decreases reported across studies. Dashed lines indicate hypothesized or indirect associations requiring further validation. Abbreviations: PSD, postoperative sleep disturbance; HPA, hypothalamic–pituitary–adrenal; IL-6, interleukin-6; TNF-α, tumor necrosis factor-alpha; CRP, C-reactive protein; BDNF, brain-derived neurotrophic factor; SCFA, short-chain fatty acids; LPS, lipopolysaccharide.

**FIGURE 2:**
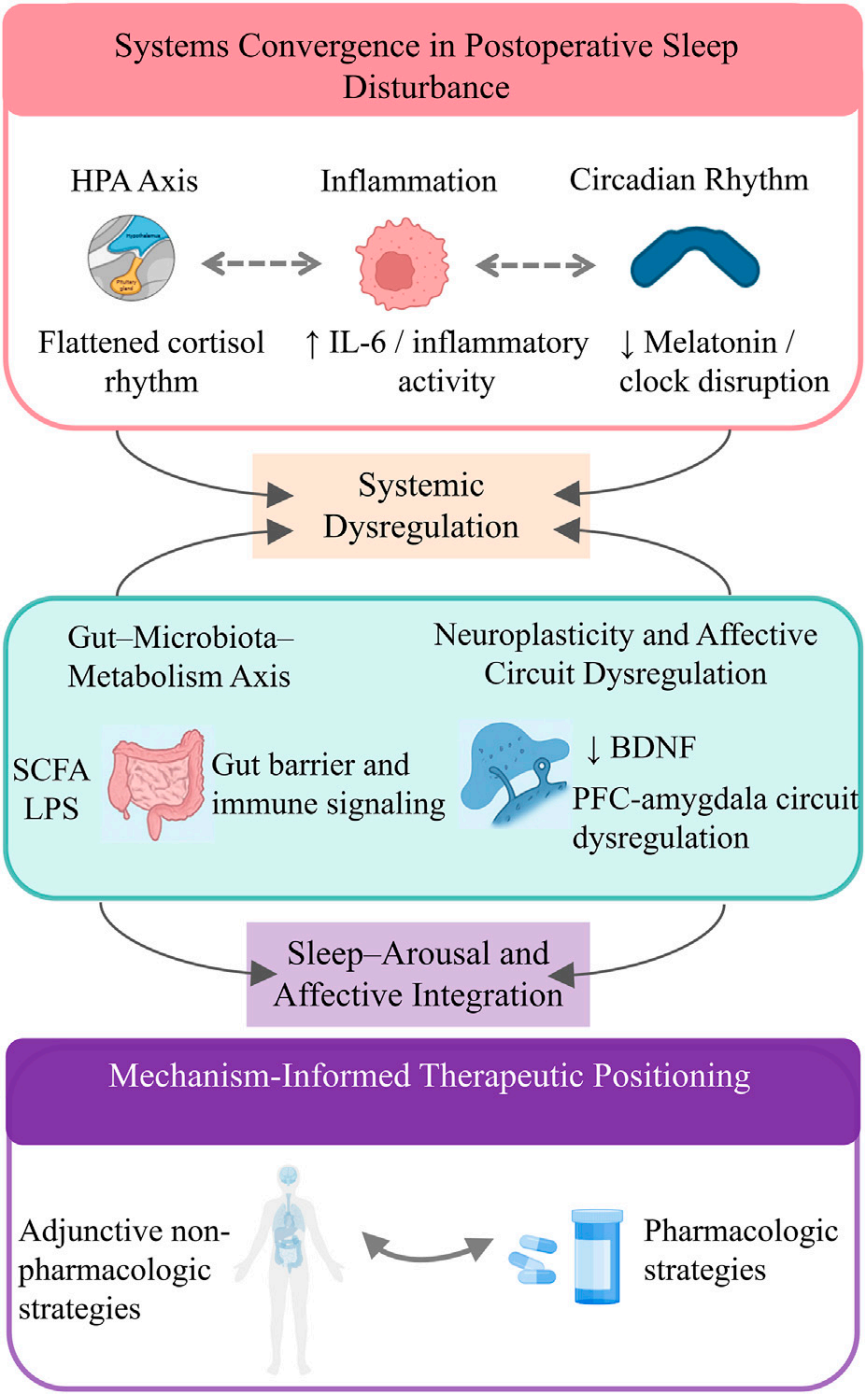
Systems convergence and mechanism-informed therapeutic positioning in postoperative sleep disturbance after breast cancer surgery. This figure illustrates a systems-level convergence model of postoperative sleep disturbance after breast cancer surgery and its implications for mechanism-informed therapeutic positioning. PSD arises from interactions among multiple biological systems, including HPA axis dysregulation, inflammatory activation, circadian rhythm disruption, autonomic imbalance, gut–microbiota–brain signaling, and neuroplasticity remodeling. These pathways converge on central sleep–wake regulation, arousal control, pain sensitization, and affective integration, leading to heterogeneous clinical phenotypes such as hyperarousal-dominant insomnia, circadian-disruption phenotypes, and pain-dominant or mixed symptom-cluster presentations. Based on this framework, the figure highlights phenotype-guided therapeutic strategies, including pharmacologic interventions (e.g., dual orexin receptor antagonists, melatonergic agents, multimodal analgesia, and gabapentinoids) alongside adjunctive behavioral and circadian-oriented approaches such as cognitive behavioral therapy for insomnia and bright light therapy. Notes: This schematic is intended to illustrate conceptual integration of biological mechanisms and phenotype-guided therapeutic positioning rather than a linear causal pathway. Abbreviations: PSD, postoperative sleep disturbance; HPA, hypothalamic–pituitary–adrenal; IL-6, interleukin-6; BDNF, brain-derived neurotrophic factor; SCFA, short-chain fatty acids; LPS, lipopolysaccharide; PFC, prefrontal cortex.

PSD after breast cancer surgery is best understood as the product of interacting biological systems rather than as an isolated postoperative complaint. Surgical stress, inflammatory activation, neuroendocrine dysregulation, circadian disruption, autonomic imbalance, and gut-microbiota-related signaling may converge within a neuroimmune-endocrine network (Figure 1), with downstream systems integration and therapeutic implications illustrated in Figure 2 (Li et al., 2024). From a translational pharmacology perspective, these interacting mechanisms are clinically relevant because they may shape symptom phenotype, influence treatment response, and help identify candidate pathways for targeted intervention.

### 4.1 Surgical stress and inflammatory activation

Breast cancer surgery induces an acute stress response characterized by tissue injury, nociceptive input, sympathetic activation, and the release of proinflammatory mediators such as interleukin-6 (IL-6) and tumor necrosis factor-alpha (TNF-alpha). Clinical findings suggest that the sympathetic-inflammatory axis is relevant to PSD pathogenesis. In women undergoing modified radical mastectomy, linearly polarized light irradiation near the stellate ganglion improved postoperative sleep and reduced IL-6 and TNF-alpha while increasing nocturnal melatonin secretion (Weiming et al., 2025). Longitudinal data from the CANTO cohort further support an association between higher IL-6 levels and subsequent fatigue and insomnia burden (Di Meglio et al., 2024). Together, these findings support perioperative inflammation as a plausible therapeutic target linking nociception, immune activation, and sleep disruption (Yang et al., 2023).

### 4.2 Dysregulation of the HPA axis

Dysregulation of the hypothalamic-pituitary-adrenal (HPA) axis may increase vulnerability to persistent PSD in women with breast cancer (Feng et al., 2023). Clinical studies have identified altered cortisol-related measures in subsets of survivors with persistent symptoms (Shardool et al., 2023). Although direct postoperative evidence remains limited, cortisol-related measures may help identify patients at higher risk for persistent PSD and may prove useful in future risk stratification or biomarker-informed intervention models (Adrian et al., 2024; Margherita et al., 2011).

### 4.3 Core neuroimmune mechanisms and biomarkers

Among candidate biomarkers relevant to PSD in breast cancer, IL-6 and C-reactive protein (CRP) currently appear the most clinically relevant, as higher levels have been associated with poorer sleep efficiency, greater fatigue, and broader symptom burden in breast cancer populations (Julienne et al., 2025). These observations support the broader concept that PSD may reflect sustained neuroimmune activation and low-grade systemic inflammation rather than sleep disturbance alone. In perioperative settings, inflammatory signaling may amplify hyperarousal, pain sensitivity, and impaired sleep continuity, thereby linking immune activation to clinically meaningful postoperative symptoms (Prather, 2022).

From a translational standpoint, biomarker-informed models may help refine therapeutic targeting in future studies. IL-6 and CRP are particularly attractive because they are clinically accessible and may serve both as mechanistic indicators and as potential response markers in trials evaluating pharmacologic, behavioral, or exercise-based interventions (Duivon et al., 2024). At present, however, the evidence remains stronger for association than causation, and biomarker-guided treatment strategies for postoperative PSD after breast cancer surgery still require prospective validation (Di Meglio et al., 2024).

### 4.4 Melatonin, circadian rhythm disruption, and cancer

Melatonin plays a central role in circadian synchronization and sleep-wake regulation. In breast cancer, circadian disruption related to surgery, hospitalization, treatment timing, nocturnal care interruption, and psychological stress may impair melatonergic signaling and delay sleep recovery. This is therapeutically relevant because circadian instability may contribute not only to insomnia, but also to fatigue, mood disturbance, and broader impairment of postoperative recovery (Motehaver et al., 2025).

From a pharmacologic and translational perspective, circadian and melatonergic pathways are especially attractive because they map directly to clinically actionable interventions, including melatonin, melatonin receptor agonists, bright light therapy, and behavioral timing strategies. These approaches may be particularly relevant in patients with delayed sleep timing, irregular sleep-wake schedules, or hospital-related circadian misalignment. Although breast cancer-specific postoperative evidence remains variable, circadian mechanisms provide one of the clearest links between pathophysiology and practical intervention (Motehaver et al., 2025).

### 4.5 The gut microbiota-brain axis and sleep

The gut microbiota-brain axis has emerged as a plausible contributor to sleep and inflammatory homeostasis, although direct evidence in postoperative breast cancer populations remains limited. Experimental and observational studies suggest that sleep loss and circadian disruption may alter microbial composition, impair intestinal barrier integrity, and promote inflammatory signaling, thereby reinforcing central sleep-wake dysregulation. In breast cancer survivorship, these interactions may also intersect with metabolic and endocrine disturbances (Schettini et al., 2024).

At present, microbiota-related mechanisms should be regarded as promising but investigational rather than clinically established drivers of PSD management (Laborda-Illanes et al., 2025). Their principal translational relevance lies in the possibility that future biomarker-stratified studies may identify subgroups in whom circadian, dietary, melatonergic, or broader microbiota-directed interventions contribute to symptom improvement.

The major mechanistic domains, representative biomarkers, and corresponding therapeutic implications of PSD after breast cancer surgery are summarized in Table 1.

**TABLE 1:** Key mechanistic mediators in postoperative sleep disturbance (PSD): sources, targets, and mechanistic effects.

Pathophysiological domain	Key biological mechanisms	Representative biomarkers/mechanistic mediators	Clinical relevance to PSD	Therapeutic implications
Surgical stress and inflammatory activation	Tissue injury, nociceptive input, sympathetic activation, and acute postoperative inflammatory responses promote sleep fragmentation and hyperarousal	IL-6, TNF-α, IL-1β, CRP	Early postoperative insomnia, non-restorative sleep, pain-related arousals, fatigue	Multimodal analgesia, anti-inflammatory pharmacologic strategies, perioperative stress reduction, selected adjunctive interventions such as acupuncture
HPA axis dysregulation	Surgical and psychological stress disrupt glucocorticoid rhythmicity and impair feedback regulation of inflammation	Multi-timepoint cortisol, DHEAS, ACTH	Hyperarousal, circadian instability, persistent insomnia, fatigue, affective symptoms	Chronobiologic interventions, CBT-I, mindfulness-based therapies, circadian restoration strategies
Core neuroimmune activation	Sustained low-grade inflammation and neuroimmune signaling amplify pain sensitization and dysregulate sleep–wake control	IL-6, CRP, TNF-α	Reduced sleep efficiency, fatigue, broader symptom burden	Exercise-based rehabilitation, anti-inflammatory strategies, biomarker-guided symptom monitoring
Circadian and melatonergic disruption	Altered melatonin secretion and circadian desynchronization related to hospitalization, treatment timing, and stress impair sleep initiation and maintenance	Melatonin rhythm, sleep–wake timing measures	Delayed sleep onset, fragmented sleep, fatigue, mood disturbance	Melatonin or melatonin receptor agonists, bright light therapy, CBT-I, circadian-timed behavioral interventions
Autonomic imbalance	Increased sympathetic activity and reduced parasympathetic tone sustain physiological hyperarousal and impair nocturnal recovery	Heart rate variability, resting heart rate	Sleep fragmentation, anxiety, impaired recovery, pain amplification	Relaxation-based interventions, mindfulness, exercise, acupuncture, selected neuromodulatory strategies
Gut–microbiota–brain axis dysfunction	Circadian disruption and sleep loss induce dysbiosis, impair intestinal barrier integrity, and enhance inflammatory signaling	Microbiome composition, short-chain fatty acids, lipopolysaccharide-related markers	Sleep instability, inflammatory burden, vulnerability to pain and anxiety	Dietary optimization, probiotics/prebiotics, circadian restoration, microbiome-directed investigational strategies
Neural plasticity and affective circuit remodeling	Persistent stress and insomnia alter limbic–prefrontal circuitry involved in arousal regulation and emotional processing	BDNF, neuroimaging-based functional connectivity markers	Chronic insomnia, affective symptoms, symptom amplification, reduced resilience	CBT-I, mindfulness-based interventions, exercise, acupuncture, restorative behavioral therapies

This table summarizes the major biological mechanisms implicated in postoperative sleep disturbance after breast cancer surgery, including key pathophysiological domains, representative biomarkers or mediators, their clinical relevance, and corresponding therapeutic implications. It provides a translational framework linking mechanistic pathways to clinically actionable intervention strategies.

Abbreviations: PSD, postoperative sleep disturbance; IL, interleukin; TNF-α, tumor necrosis factor-alpha; CRP, C-reactive protein; HPA, hypothalamic–pituitary–adrenal; DHEAS, dehydroepiandrosterone sulfate; ACTH, adrenocorticotropic hormone; BDNF, brain-derived neurotrophic factor.

## 5 Current therapeutic management of PSD in breast cancer

Management of PSD after breast cancer surgery should be guided by symptom phenotype, severity, treatment context, and safety considerations. In current practice, pharmacologic management remains the principal therapeutic strategy for acute or clinically significant postoperative insomnia, particularly when nocturnal hyperarousal, pain-related sleep disruption, or mixed symptom clusters are prominent. Accordingly, PSD should be approached not only as a symptom-management problem but also as a target for mechanism-informed therapeutic selection (Nissen et al., 2024). Non-pharmacologic and integrative interventions are best positioned as adjunctive or maintenance-oriented strategies that support durability of benefit and reduce long-term sedative burden rather than define primary treatment.

Table 2 summarizes pharmacologic approaches and breast cancer-specific cautions, while Table 3 summarizes adjunctive strategies.

**TABLE 2:** Pharmacologic therapies for postoperative sleep disturbance (PSD): mechanisms, sleep benefits, and oncology-specific cautions.

Drug class	Pharmacologic mechanism/target	Clinical positioning in PSD	Key breast cancer-specific safety considerations
Benzodiazepines/Z-drugs	Positive allosteric modulation of GABA_A receptors; suppression of arousal-promoting neural circuits	Short-term control of acute hyperarousal-related insomnia; rapid sleep initiation and maintenance	Use extreme caution with concurrent opioids because of additive sedation and respiratory depression; associated with dependence, cognitive impairment, falls, and next-day psychomotor impairment
Melatonin and melatonin receptor agonists	MT1/MT2 receptor activation; circadian phase realignment	Best suited for circadian-disruption phenotypes, delayed sleep onset, and hospital-associated sleep–wake misalignment	Variable efficacy depending on circadian disruption severity and environmental light exposure; limited long-term postoperative data
Antidepressants (SSRIs/SNRIs/SARIs)	Serotonergic and noradrenergic modulation; in selected agents, 5-HT₂A antagonism and sedative effects	Most appropriate when insomnia coexists with anxiety, depression, or affective distress	Strong CYP2D6 inhibitors (e.g., paroxetine, fluoxetine) may reduce tamoxifen efficacy; trazodone may be considered in selected insomnia-predominant cases
Atypical antipsychotics	Multi-receptor antagonism involving 5-HT₂A, H1, and D2 receptors	Reserved for refractory insomnia with coexisting affective or psychiatric symptoms	Risk of metabolic syndrome, QT prolongation, extrapyramidal symptoms, and excessive sedation; long-term use solely for insomnia is discouraged
Dual orexin receptor antagonists (DORAs)	OX1/OX2 receptor blockade; inhibition of hypothalamic wake-promoting signaling	Particularly relevant for hyperarousal-dominant insomnia and sleep-maintenance difficulty	Limited direct evidence in postoperative oncology populations; monitor for daytime somnolence and additive CNS depression with concurrent sedatives
Gabapentinoids	Binding to the α₂δ subunit of voltage-gated calcium channels; reduced excitatory neurotransmitter release	Most suitable for pain-dominant or mixed symptom-cluster phenotypes with nocturnal pain-related arousals	Increased risk of respiratory depression with opioids; dizziness, gait instability, and renal dose adjustment required
Cannabinoids/endocannabinoid modulators	CB1/CB2 receptor modulation; effects on pain, arousal, and inflammatory signaling	Potential adjunctive role in selected patients with pain-related sleep disturbance	Potential pharmacokinetic and pharmacodynamic interactions with endocrine or targeted therapies; limited direct evidence in postoperative breast cancer cohorts
Antihistamines/OTC sedatives	Central H1 receptor blockade with anticholinergic effects	Short-term rescue use for transient insomnia only	Cognitive impairment, residual daytime sedation, anticholinergic burden, and tolerance risk, particularly in older or chemotherapy-treated survivors

This table summarizes the principal pharmacologic therapies currently used or considered for postoperative sleep disturbance after breast cancer surgery, highlighting mechanisms of action, expected sleep-related benefits, and breast cancer-specific safety considerations, including endocrine therapy relevance and risks related to polypharmacy.

Abbreviations: GABA-A, gamma-aminobutyric acid type A; MT1/MT2, melatonin receptor 1/2; SSRI, selective serotonin reuptake inhibitor; SNRI, serotonin–norepinephrine reuptake inhibitor; SARI, serotonin antagonist and reuptake inhibitor; 5-HT, 5-hydroxytryptamine; CYP2D6, cytochrome P450 2D6; DORA, dual orexin receptor antagonist; OX1/OX2, orexin receptor 1/2; CB1/CB2, cannabinoid receptor 1/2; OTC, over-the-counter; H1, histamine-1, receptor.

**TABLE 3:** Adjunctive non-pharmacologic and integrative strategies for postoperative sleep disturbance after breast cancer surgery.

Strategy	Principal role in PSD management	Best-fit clinical context	Main strengths	Key limitations/Safety considerations	Current positioning in this review
Cognitive behavioral therapy for insomnia (CBT-I)	Core behavioral intervention; supports durable sleep improvement and medication de-escalation	Persistent insomnia beyond the acute postoperative phase; patients at risk of long-term sedative use	Strongest evidence base among non-pharmacologic interventions; improves insomnia severity, sleep quality, and may benefit fatigue and mood	Limited access to trained providers; slower onset compared with pharmacologic therapy in acute distress	First-line non-pharmacologic adjunct; central role in long-term management and sedative reduction
Bright light therapy (BLT)	Circadian-oriented adjunct	Circadian misalignment, delayed sleep phase, irregular sleep–wake patterns, hospital-associated disruption	Mechanism-aligned for circadian phenotypes; complements melatonin and behavioral timing strategies	Optimal timing, intensity, and duration not fully standardized; limited direct evidence in postoperative breast cancer populations	Selective adjunct, particularly when circadian disruption predominates
Exercise-based rehabilitation/yoga/mindfulness-based interventions	Supportive symptom-cluster management	Patients with fatigue, psychological distress, reduced physical function, or broader survivorship burden	Multidimensional benefits (fatigue, mood, physical recovery); improves overall wellbeing	Effects on sleep are indirect and less specific; not sufficient as stand-alone therapy for acute insomnia	Supportive adjuncts; not primary insomnia treatments
Digital CBT-I and wearable-supported monitoring	Delivery and monitoring platforms	Limited access to in-person CBT-I; patients suitable for remote care and longitudinal tracking	Scalable access; enhances adherence, follow-up, and sleep monitoring	Wearables are assessment tools rather than treatments; oncology-specific long-term efficacy remains under evaluation	Adjunctive tools for delivery, monitoring, and adherence support
Acupuncture/electroacupuncture/auricular therapy	Integrative adjunct for symptom clusters	Persistent insomnia with coexisting pain, fatigue, or emotional distress despite standard care	Most developed evidence among integrative approaches; may benefit multi-symptom burden	Heterogeneity in protocols; variable evidence quality; limited breast cancer-specific postoperative data	Preferred integrative option in selected patients
Chinese herbal medicine (CHM)	Investigational integrative therapy	Selected patients under specialist supervision	Potential symptom-modifying effects in survivorship settings	Limited direct evidence for PSD; variability in formulations; concerns regarding standardization and herb–drug interactions	Investigational; not recommended for routine use
Microbiota-directed interventions	Exploratory translational approach	Research settings; biomarker-driven or hypothesis-generating studies	Mechanistic relevance to inflammation, circadian regulation, and gut–brain signaling	Insufficient clinical evidence; no established therapeutic role	Investigational; currently limited to research contexts
Moxibustion and other complementary modalities	Supportive complementary interventions	Selected patients within integrative or rehabilitation programs	May provide symptomatic relief in some contexts	Limited and heterogeneous evidence; not adequate as stand-alone insomnia therapy	Selective adjunct only

This table summarizes adjunctive behavioral, circadian-oriented, and integrative strategies for postoperative sleep disturbance, with emphasis on their principal clinical roles, best-fit contexts, strengths, limitations, and current positioning within the present review.

Abbreviations: PSD, postoperative sleep disturbance; CBT-I, cognitive behavioral therapy for insomnia; BLT, bright light therapy; CHM, chinese herbal medicine.

### 5.1 Pharmacologic interventions

Pharmacologic treatment remains the principal clinical strategy for managing PSD in women with breast cancer, particularly when insomnia is acute, functionally impairing, pain-associated, or accompanied by prominent anxiety or treatment-related distress. Major drug classes include benzodiazepines and non-benzodiazepine hypnotics, melatonin and melatonin receptor agonists, selected antidepressants and antipsychotics, dual orexin receptor antagonists (DORAs), gabapentinoids, cannabinoid-based agents, and sedating antihistamines. In breast cancer survivorship, treatment selection should be guided by symptom phenotype, endocrine therapy context, comorbidity burden, anticipated duration of use, and the risk of respiratory or cognitive adverse effects (Grassi et al., 2023). A phenotype-guided and safety-informed prescribing framework may therefore help organize treatment selection, but it should currently be regarded as a proposed clinical framework rather than a validated prescribing algorithm.

Across drug classes, safety should remain central to treatment selection. Concurrent use of opioids with benzodiazepines, Z-drugs, sedating antihistamines, or gabapentinoids may substantially increase the risk of oversedation, respiratory compromise, falls, and delayed recovery, particularly in older or medically complex patients. Drug choice should also account for endocrine therapy context, including avoidance of strong CYP2D6 inhibitors in patients receiving tamoxifen, as well as potential duplicate sedative exposure from over-the-counter sleep aids. In general, the safest approach is to use the lowest effective dose for the shortest necessary duration, with regular reassessment of ongoing indication and cumulative medication-related risk (Saeki et al., 2024).

#### 5.1.1 Benzodiazepines and Z-drugs

Benzodiazepines and non-benzodiazepine hypnotics are widely prescribed for sleep disturbance in women with breast cancer, particularly during chemotherapy and the early survivorship period. Cohort studies indicate that up to 30% of patients initiate sedative-hypnotic therapy during adjuvant chemotherapy, and persistent use may occur in a substantial proportion thereafter (Jacob et al., 2022; Moore et al., 2011). These agents enhance GABA_A receptor signaling and facilitate sleep onset and maintenance. Z-drugs, including zolpidem, zaleplon, and eszopiclone, show relative selectivity for the α1 subunit of the GABA_A receptor and therefore produce predominantly hypnotic effects with somewhat less residual sedation.

However, chronic use of benzodiazepines and Z-drugs is associated with tolerance, cognitive impairment, falls, and potential respiratory depression, especially when combined with opioids in postoperative, older, or frail patients. Polysomnographic studies in breast cancer survivors further indicate that sleep fragmentation and periodic limb movement burden may persist despite symptomatic hypnotic treatment, suggesting that sleep architecture abnormalities are not fully corrected by these agents (Ruth et al., 2015). In breast cancer survivorship, these drugs are best positioned as short-term rescue options for acute insomnia with prominent nocturnal hyperarousal rather than as long-term treatment strategies.

#### 5.1.2 Melatonin and melatonin receptor agonists

Melatonin is a pineal hormone essential for circadian synchronization and sleep-wake regulation. In women with breast cancer, surgery, chemotherapy, radiotherapy, and hospital-related circadian disruption may alter melatonin secretion and contribute to sleep instability (Hanan et al., 2020). Exogenous melatonin and selective melatonin receptor agonists such as ramelteon therefore represent biologically plausible pharmacologic options for PSD, particularly in patients with circadian misalignment, delayed sleep timing, or reduced nocturnal melatonergic signaling. A meta-analysis of five studies involving 243 breast cancer patients reported significant improvement in sleep quality and reduced insomnia severity with melatonin-containing regimens (Kyoungsan et al., 2023).

Melatonin acts mainly through MT1 and MT2 receptors to support sleep onset and circadian alignment (Alev et al., 2021). From a clinical positioning perspective, melatonergic therapy may be particularly useful when circadian disruption is a dominant driver of PSD and when a safer alternative to traditional sedative-hypnotics is desirable.

#### 5.1.3 Antidepressants and antipsychotics

Anxiety and depression commonly coexist with insomnia in women with breast cancer. Selective serotonin reuptake inhibitors (SSRIs) and serotonin-norepinephrine reuptake inhibitors (SNRIs) are therefore frequently prescribed to address affective symptoms and may secondarily improve sleep by reducing distress and hyperarousal. However, antidepressant selection requires particular caution in patients receiving tamoxifen, because strong cytochrome P450 2D6 (CYP2D6) inhibitors such as paroxetine, fluoxetine, and bupropion may reduce conversion of tamoxifen to its active metabolite endoxifen and potentially compromise endocrine therapy efficacy (Keller et al., 2026). In this context, citalopram, escitalopram, venlafaxine, and low-dose trazodone are generally considered more appropriate options.

Trazodone, a serotonin antagonist and reuptake inhibitor, promotes sleep mainly through antagonism of 5-HT_2_A and histamine H_1_ receptors together with mild α_1_-adrenergic blockade, thereby reducing cortical arousal and prolonging non-rapid eye movement sleep (Andrea et al., 2025). Chronic use may result in next-day sedation, dizziness, and orthostatic symptoms.

Atypical antipsychotics such as quetiapine and olanzapine are occasionally used for refractory insomnia because of combined antagonism of 5-HT_2_A, H_1_, and dopamine D_2_ receptors, which can dampen hyperarousal and improve sleep continuity. However, these agents are associated with substantial metabolic adverse effects, including weight gain, insulin resistance, and dyslipidemia, and their long-term use solely for insomnia in breast cancer survivors is generally discouraged (Šoukalová, 2022).

In clinical practice, these agents should be reserved for carefully selected patients in whom refractory insomnia coexists with affective or treatment-related indications, and they should be chosen primarily according to coexisting psychiatric symptoms and endocrine treatment context rather than as routine first-line hypnotic therapy.

#### 5.1.4 Orexin receptor antagonists (DORAs)

Dual orexin receptor antagonists, including suvorexant, lemborexant, and daridorexant, represent a newer class of hypnotics that reduce wake drive by blocking orexin signaling (Isabelle et al., 2020). Unlike benzodiazepines or Z-drugs, DORAs do not directly potentiate GABAergic transmission and may therefore improve sleep initiation and maintenance while more closely preserving physiological sleep architecture and reducing dependence-related concerns.

Clinical trials in primary insomnia populations have demonstrated improvements in sleep onset and maintenance with generally favorable tolerability; however, direct breast cancer-specific evidence remains very limited, and there are currently no completed randomized controlled trials that definitively establish the efficacy or safety of DORAs for postoperative sleep disturbance after breast cancer surgery. Available data do not suggest a major excess risk of falls or fractures relative to other hypnotics, although caution remains necessary in frail patients (Pan et al., 2024). In the context of PSD, DORAs should therefore be considered mechanistically plausible but investigational options for hyperarousal-related insomnia, and their use in breast cancer survivorship should be approached cautiously because of limited direct evidence, polypharmacy concerns, CYP3A-mediated interaction potential, fall risk, fatigue, and concurrent opioid exposure.

#### 5.1.5 Gabapentinoids

Gabapentinoids, including gabapentin and pregabalin, bind to the α_2_δ subunit of voltage-gated calcium channels, reduce neuronal hyperexcitability, and may lessen pain-related arousal. Clinical studies in neuropathic pain populations suggest improvement in sleep quality and reduced pain-related sleep interference, supporting their relevance in symptom clusters in which pain is a major driver of insomnia (Daniel et al., 2019).

In breast cancer care, gabapentin and pregabalin are used off-label for perioperative pain, chemotherapy-induced neuropathic symptoms, and nocturnal discomfort, with secondary benefits on sleep and anxiety in some patients. However, their use is limited by dizziness, somnolence, gait instability, and potential misuse, particularly in vulnerable survivors or at higher doses (Angela et al., 2024). Concomitant use with opioids further increases the risk of respiratory depression, especially in older patients or those with multimorbidity, making cautious dosing, renal adjustment, and close monitoring essential (Mrinmayee et al., 2023). Accordingly, gabapentinoids are best positioned as adjunctive pharmacologic agents in pain-dominant or neuropathic symptom phenotypes rather than as routine first-line hypnotics for PSD.

#### 5.1.6 Cannabinoids and endocannabinoid modulators

Cannabinoid-based therapies may influence pain and related symptom burden, but evidence for PSD in breast cancer remains limited (Dobovišek et al., 2020). Most available data are extrapolated from mixed oncology populations, and concerns remain regarding variable product quality, neurocognitive adverse effects, sedation, and potential pharmacokinetic or pharmacodynamic interactions with endocrine or targeted therapies (Deng and Li, 2020; Almeida et al., 2021). Regulatory restrictions also vary across jurisdictions. At present, cannabinoids should be regarded as carefully supervised pharmacologic adjuncts in selected patients rather than routine therapies for PSD after breast cancer surgery.

#### 5.1.7 Antihistamines and OTC sedatives

Sedating antihistamines such as diphenhydramine, doxylamine, and hydroxyzine are commonly used for transient insomnia among women undergoing and recovering from breast cancer treatment, including during adjuvant chemotherapy (Moore et al., 2011). These agents promote sleep primarily through central H_1_ receptor blockade. Off-target antimuscarinic effects may contribute to sedation but also lead to dry mouth, constipation, urinary retention, blurred vision, and cognitive slowing.

Although sedating antihistamines may shorten sleep latency, they frequently cause residual daytime drowsiness, tolerance, and impaired attention, particularly in older or heavily treated survivors (Panel, 2023). Long-term efficacy data are limited, and chronic use may worsen fatigue and increase fall risk, making these drugs more suitable for short-term or rescue use than for ongoing management of PSD. Real-world data further suggest extensive use of non-prescription products, including over-the-counter medicines and herbal or lifestyle remedies, among breast cancer survivors receiving endocrine therapy for management of vasomotor symptoms and associated sleep disturbance (Kingsberg et al., 2024). Because diphenhydramine-containing nighttime formulations and multi-ingredient sedative products may contribute to anticholinergic burden or interact with endocrine and targeted therapies through cytochrome P450 pathways, routine assessment of over-the-counter sleep-aid use is important in survivorship care. These agents are better viewed as short-term rescue options than as sustainable therapeutic strategies for PSD.

#### 5.1.8 Multimodal analgesia and perioperative symptom-cluster management

Pain is a major driver of PSD after breast cancer surgery and should be considered a core therapeutic target rather than a parallel symptom. In the perioperative setting, insomnia, nocturnal arousal, pain, anxiety, opioid exposure, and inflammatory activation frequently coexist as an interconnected symptom cluster. Accordingly, multimodal analgesia is highly relevant to PSD management because it may improve sleep indirectly by reducing nociceptive input, limiting nighttime awakenings, decreasing opioid requirements, and attenuating postoperative physiological stress (Carson et al., 2026).

In clinical practice, multimodal analgesia may include non-opioid analgesics, regional or local anesthetic techniques, perioperative anti-inflammatory strategies, and selected adjunctive agents for neuropathic or mixed pain phenotypes. These approaches are particularly important in breast cancer populations because pain presentations are heterogeneous and may include incisional pain, neuropathic symptoms, chest wall discomfort, drain-related irritation, and movement-associated pain. Patients with pain-dominant insomnia may therefore benefit less from escalation of conventional hypnotics alone and more from integrated symptom-cluster management in which analgesic optimization and sleep-directed therapy are coordinated.

From a safety perspective, opioid-sparing strategies are especially important because concurrent use of opioids with benzodiazepines, Z-drugs, sedating antihistamines, or gabapentinoids may increase the risk of oversedation, respiratory compromise, falls, and delayed functional recovery. Overall, multimodal analgesia should be regarded as a central pharmacologic component of PSD management in patients with pain-dominant or mixed symptom presentations.

#### 5.1.9 Safety considerations across drug classes

Across pharmacologic options for PSD, safety should remain central to treatment selection. In the perioperative setting, cumulative sedative burden from concurrent opioids, benzodiazepines, Z-drugs, sedating antihistamines, or gabapentinoids may increase the risk of oversedation, respiratory compromise, falls, and delayed functional recovery, particularly in older or medically complex patients (Institute, 2016). Cognitive adverse effects, next-day impairment, gait instability, and anticholinergic burden are also clinically important (Sychev et al., 2024). In breast cancer survivorship, drug choice should additionally account for breast cancer-specific treatment context. In patients receiving tamoxifen, strong CYP2D6 inhibitors should be avoided when possible because they may reduce conversion of tamoxifen to its active metabolite endoxifen. Aromatase inhibitors may contribute to insomnia indirectly through arthralgia, musculoskeletal discomfort, vasomotor symptoms, and mood disturbance, suggesting that symptom-targeted management may be preferable to nonspecific hypnotic escalation in selected patients. CDK4/6 inhibitors require attention to CYP3A-mediated interactions, and ribociclib additionally raises concern regarding QT interval prolongation when combined with other QT-prolonging agents. HER2-targeted therapies and chemotherapy may worsen sleep through fatigue, nausea, neuropathy, cardiotoxicity-related concerns, and treatment-related distress. Corticosteroids used as antiemetic or chemotherapy-related supportive medications may cause sleep-onset insomnia, particularly when administered later in the day. These breast cancer-specific treatment factors highlight the need for individualized pharmacologic selection rather than a uniform hypnotic-centered approach. In general, treatment should use the lowest effective dose for the shortest necessary duration, with regular reassessment of ongoing indication and cumulative medication-related risk (Sun et al., 2025).

### 5.2 Adjunctive non-pharmacologic and integrative strategies

Non-pharmacologic and integrative approaches should be positioned primarily as adjunctive or maintenance-oriented strategies rather than substitutes for pharmacologic management in PSD after breast cancer surgery. Their principal clinical value lies in improving durability of benefit, addressing coexisting fatigue, anxiety, and circadian disruption, and supporting reduction of long-term sedative burden.

Among behavioral interventions, cognitive behavioral therapy for insomnia (CBT-I) has the strongest evidence base and is best regarded as the leading non-pharmacologic adjunct, particularly for insomnia that persists beyond the immediate postoperative period (Ma et al., 2021). In breast cancer populations, CBT-I has shown meaningful improvements in sleep quality and insomnia severity, with additional benefits for fatigue and affective symptoms in some studies (Greeley et al., 2025). In practice, CBT-I is especially valuable as a medication-sparing and de-escalation strategy.

Other supportive approaches may be considered according to symptom profile and feasibility. Bright light therapy may be useful when circadian disruption or irregular sleep-wake timing is prominent, particularly as an adjunct to melatonergic or behavioral interventions (Yao et al., 2023). Exercise-based rehabilitation, yoga, and mindfulness-oriented approaches may provide broader symptom-cluster benefits, although these should be viewed as supportive rather than primary insomnia treatments (Ee et al., 2024). Digital CBT-I and wearable-supported monitoring may improve access and longitudinal management but currently function mainly as delivery or monitoring tools rather than stand-alone therapies (Starling et al., 2024).

Integrative approaches should be used selectively. Among these, acupuncture currently has the most developed evidence base and may be considered in carefully selected patients with persistent insomnia accompanied by pain, fatigue, or emotional distress despite standard management (Yang et al., 2025). By contrast, Chinese herbal medicine, microbiota-directed interventions, moxibustion, and related complementary modalities remain investigational because of limited direct evidence, heterogeneity of interventions, and unresolved concerns regarding standardization, safety, and interactions with ongoing cancer therapies.

Overall, within a clinically relevant pharmacologic framework, non-pharmacologic and integrative strategies are best incorporated as adjuncts that complement phenotype-guided pharmacologic management rather than replace it.

### 5.3 Clinical positioning and phenotype-guided management

Taken together, the available evidence supports the rationale for a stepwise, phenotype-guided approach to PSD after breast cancer surgery, although direct clinical validation of this approach remains limited (Kim et al., 2026). Pharmacologic management remains central for acute or clinically significant insomnia, but treatment selection should be individualized according to the dominant drivers of sleep disturbance, including nocturnal hyperarousal, pain-related arousal, circadian misalignment, affective distress, and persistence of symptoms beyond the immediate postoperative period. Within this framework, non-pharmacologic and integrative approaches are best positioned as adjunctive or maintenance-oriented strategies that improve durability of benefit and help reduce long-term medication burden.

In patients with acute postoperative insomnia characterized by prominent nocturnal hyperarousal and difficulty initiating sleep, short-course hypnotic therapy may be appropriate when respiratory risk, cognitive vulnerability, and concurrent opioid exposure are carefully considered (Ji and Liu, 2025). When circadian disruption or hospital-related sleep-wake misalignment predominates, melatonin or melatonin receptor agonists may offer a more mechanism-aligned option, with adjunctive use of bright light therapy or behavioral timing strategies where feasible. By contrast, patients with pain-dominant or mixed insomnia presentations may benefit more from optimization of multimodal analgesia and selective use of agents such as gabapentinoids than from escalation of conventional hypnotics alone.

For insomnia that persists beyond the acute postoperative phase or extends into survivorship, CBT-I should be considered the leading behavioral adjunct and the preferred strategy for improving longer-term sleep regulation and reducing reliance on sedative medications. Exercise-based rehabilitation, mindfulness-based approaches, and circadian-oriented interventions may be added according to symptom profile, treatment tolerance, and patient preference (Da Silva et al., 2024).

Across phenotypes, treatment selection should remain closely linked to cumulative sedative burden, opioid exposure, endocrine therapy context, and overall safety profile.

### 5.4 Practical clinical management framework

**FIGURE 3:**
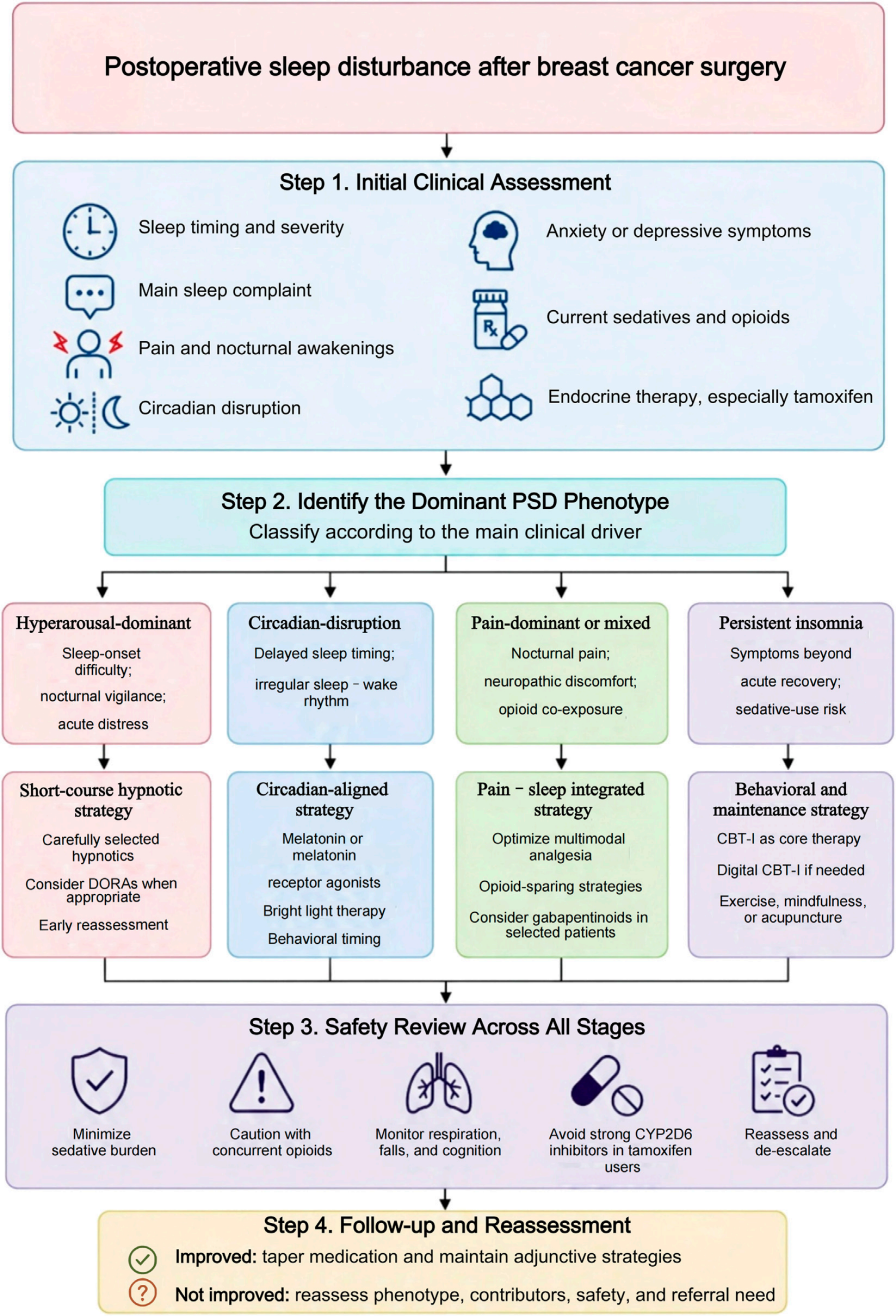
Practical clinical flowchart for phenotype-guided management of postoperative sleep disturbance after breast cancer surgery. This flowchart summarizes a stepwise clinical approach for the assessment and management of postoperative sleep disturbance (PSD) after breast cancer surgery. Initial assessment includes sleep timing and severity, predominant sleep complaint, pain burden and nocturnal awakenings, circadian disruption, anxiety or depressive symptoms, current sedative and opioid exposure, and endocrine therapy context, especially tamoxifen use. Patients may then be stratified according to the dominant clinical phenotype, including hyperarousal-dominant, circadian-disruption, pain-dominant or mixed, and persistent insomnia phenotypes. Corresponding management strategies include short-course hypnotic therapy for acute hyperarousal-related insomnia, circadian-aligned strategies such as melatonin or melatonin receptor agonists and bright light therapy, pain–sleep integrated strategies including multimodal analgesia and selected gabapentinoids, and behavioral or maintenance strategies centered on cognitive behavioral therapy for insomnia (CBT-I). Across all stages, safety review should include cumulative sedative burden, concurrent opioid exposure, respiratory risk, fall risk, cognitive impairment, and avoidance of strong CYP2D6 inhibitors in patients receiving tamoxifen. Follow-up should emphasize treatment response, medication de-escalation when feasible, reassessment of phenotype and contributing factors, and referral when symptoms remain uncontrolled. Abbreviations: PSD, postoperative sleep disturbance; DORA, dual orexin receptor antagonist; CBT-I, cognitive behavioral therapy for insomnia; CYP2D6, cytochrome P450 2D6.

From a practical perspective, management of PSD after breast cancer surgery should begin with identification of the dominant symptom phenotype rather than routine escalation of hypnotic therapy alone (Carson et al., 2026). As illustrated in Figure 2, the convergence of interacting biological systems provides a basis for mechanism-informed therapeutic positioning. To further enhance clinical applicability, Figure 3 presents a practical diagnostic and therapeutic flowchart that summarizes initial assessment, phenotype stratification, treatment selection, safety review, and follow-up for PSD after breast cancer surgery.

For patients with acute insomnia marked by short-term distress and difficulty initiating sleep, a short course of hypnotic therapy may be reasonable when symptom severity justifies pharmacologic treatment and concurrent sedative burden is acceptable (Kianian et al., 2024). When circadian misalignment is more prominent, melatonin or melatonin receptor agonists may be preferred, ideally combined with behavioral timing measures or bright light therapy. In contrast, when pain is the dominant contributor, priority should be given to multimodal analgesia, opioid-sparing strategies, and treatment of neuropathic or mixed pain phenotypes; in carefully selected patients, gabapentinoids may be considered as adjunctive agents for pain-related arousal rather than as routine first-line hypnotics (Niklasson et al., 2025).

When symptoms persist beyond the immediate postoperative period, CBT-I should be incorporated as the principal non-pharmacologic strategy to improve longer-term sleep regulation and support medication de-escalation. Exercise-based rehabilitation, mindfulness-related interventions, and digital CBT-I or wearable-supported monitoring may be added according to symptom profile, functional status, and access to care (Høeg et al., 2025).

Across all stages of management, safety review should be continuous. Clinicians should reassess cumulative sedative burden, concurrent opioid exposure, next-day cognitive impairment, fall risk, endocrine therapy context, and potential drug-drug or herb-drug interactions, including avoidance of strong CYP2D6 inhibitors in patients receiving tamoxifen. Overall, the most practical current model is a layered management pathway in which pharmacologic management provides short-term symptom control, while behavioral, circadian, analgesic, and selected adjunctive strategies are layered according to dominant symptom phenotype and recovery stage (Du et al., 2025).

## 6 Emerging pharmacologic strategies and future research directions for PSD after breast cancer surgery

Emerging strategies for PSD after breast cancer surgery extend beyond current clinical practice and aim to refine mechanism-based and phenotype-informed therapeutic development.

PSD represents a clinically important but therapeutically underdeveloped condition at the intersection of perioperative medicine, sleep pharmacology, symptom-cluster science, and oncology survivorship (Vaughn and Vaughn, 2025). Although many patients experience clinically meaningful insomnia, nocturnal hyperarousal, circadian disruption, pain-related sleep fragmentation, and persistent fatigue during recovery, current management remains largely empirical and is frequently extrapolated from primary insomnia populations or broader oncology cohorts. This limits therapeutic precision and highlights a critical gap in pharmacologic development.

From a drug development perspective, PSD should not be viewed as a transient postoperative complaint requiring nonspecific sedation. Rather, it represents a phenotype-heterogeneous and mechanism-relevant condition in which disturbed sleep arises from interacting disruptions in wake regulation, nociceptive processing, circadian timing, inflammatory signaling, and stress physiology. This conceptual framework has direct implications for pharmacologic development, supporting target-based candidate selection, phenotype-enriched trial design, and biomarker-linked evaluation of efficacy and safety (Chino et al., 2025).

### 6.1 Mechanism-informed therapeutic framework

A central translational concept in PSD after breast cancer surgery is that symptom burden is unlikely to be explained by a single pathway. Instead, postoperative insomnia and sleep fragmentation arise from convergence across interacting systems, including inflammatory signaling, hypothalamic-pituitary-adrenal axis dysregulation, autonomic arousal, circadian misalignment, and pain-related neural sensitization. Surgical tissue injury and nociceptive input may activate inflammatory and sympathetic pathways, which in turn increase physiological hyperarousal and impair sleep continuity, while disrupted sleep may further exacerbate pain sensitivity, affective distress, and circadian instability (Yin et al., 2026).

This systems-based framework helps explain why escalation of conventional hypnotics is often insufficient in postoperative breast cancer populations. Patients with predominant nocturnal hyperarousal differ clinically from those whose sleep disturbance is driven primarily by pain, circadian misalignment, or persistent treatment-related distress. Accordingly, translational progress requires moving beyond syndromic definitions toward clinically actionable phenotypes, including hyperarousal-dominant insomnia, circadian-disruption phenotypes, pain-dominant or mixed symptom-cluster phenotypes, and persistent insomnia extending into survivorship (Zhou et al., 2026).

These distinctions have direct pharmacologic implications. Hyperarousal-dominant presentations may be more suitable for wake-targeting agents, circadian-disruption phenotypes for melatonergic or chronobiologic therapies, and pain-dominant or mixed phenotypes for multimodal analgesia or selectively repurposed agents targeting symptom clusters rather than conventional hypnotics alone. In this context, mechanism-informed treatment provides a practical foundation for candidate drug selection, trial design, and interpretation of treatment response in PSD after breast cancer surgery.

### 6.2 Orexin and melatonergic pathways as emerging drug targets

Among emerging pharmacologic directions, the orexin system represents a mechanistically plausible but still clinically unvalidated target for treatment development in PSD after breast cancer surgery. Orexin signaling plays a central role in stabilizing wakefulness and sustaining arousal, and is therefore particularly relevant in postoperative insomnia characterized by persistent nocturnal wake drive driven by surgical stress, pain, anxiety, and neuroendocrine activation. Dual orexin receptor antagonists (DORAs), including suvorexant, lemborexant, and daridorexant, directly target this pathway by reducing orexin-mediated wake signaling rather than broadly potentiating GABAergic inhibition (Wu et al., 2022).

This pharmacologic profile is theoretically advantageous in postoperative breast cancer populations, but this assumption is based largely on extrapolation from primary insomnia studies rather than direct evidence from breast cancer surgery cohorts. DORAs may be theoretically relevant for patients with sleep-initiation difficulty, fragmented sleep, and physiological hyperarousal, while offering a potentially more physiologically aligned approach to sleep promotion with relative preservation of sleep architecture compared with traditional hypnotics (Di Marco et al., 2024). In addition, they may be considered in future studies for patients in whom long-term benzodiazepine or Z-drug exposure is undesirable because of risks of tolerance, cognitive impairment, falls, or dependence-related effects.

However, the role of DORAs in postoperative breast cancer PSD remains incompletely defined. Most available evidence derives from primary insomnia populations rather than perioperative oncology cohorts. Key unresolved questions include their safety in the context of concurrent opioid use, differential efficacy across symptom phenotypes, and whether improvements in sleep architecture translate into clinically meaningful outcomes such as reduced fatigue, improved daytime functioning, or attenuation of symptom-cluster burden. These gaps support further evaluation of DORAs in phenotype-stratified clinical trials in breast cancer surgery populations, but they do not currently justify routine preferential use over better-established approaches (Jiang et al., 2024). A registered trial evaluating suvorexant for insomnia in breast cancer survivors receiving endocrine therapy was terminated because of failure to reach enrollment milestones, with only limited enrollment reported, further underscoring the absence of definitive breast cancer-specific randomized evidence (Sabat et al., 2025).

Circadian and melatonergic pathways represent a second major area of therapeutic opportunity. Circadian disruption is highly plausible after breast cancer surgery because hospitalization, nocturnal care interruptions, psychological stress, altered light exposure, and perioperative pharmacologic regimens may collectively destabilize sleep-wake timing. In this context, melatonin and melatonin receptor agonists should be viewed as mechanism-aligned therapies for circadian-driven PSD rather than as nonspecific sleep aids (Gao et al., 2022).

Melatonin is inexpensive, widely available, and mechanistically relevant to both sleep initiation and circadian phase regulation, while selective melatonin receptor agonists such as ramelteon provide more standardized receptor-based targeting (Aiello et al., 2023). These agents may be particularly useful in patients with delayed sleep timing, irregular sleep-wake schedules, reduced nocturnal melatonergic signaling, or hospital-associated circadian misalignment. They may also be preferred when minimizing cognitive adverse effects or cumulative sedative burden is a priority.

From a translational perspective, the greatest opportunity may lie in integrating melatonergic therapy with broader chronobiologic strategies rather than using it in isolation (Zhang et al., 2026). Such approaches may include pharmacologic melatonergic treatment combined with structured behavioral timing, morning bright light exposure, daytime activity scheduling, and reduction of nighttime environmental disruption (Guo et al., 2023). This multimodal circadian alignment strategy is particularly relevant in breast cancer populations, in whom circadian instability often coexists with fatigue, mood disturbance, and treatment-related physiological stress.

Despite these promising directions, both orexin- and melatonergic-based strategies require direct validation in postoperative breast cancer cohorts (Madsen et al., 2016). Future studies should extend beyond short-term sleep endpoints to include sleep continuity, symptom-cluster burden, daytime functioning, treatment tolerability, cumulative sedative load, and durability after treatment discontinuation. More broadly, these approaches are best viewed not as universal replacements for conventional hypnotics, but as key components of a more selective, phenotype-aligned therapeutic model.

Importantly, their translational value lies in the alignment between biological target and clinical phenotype. Orexin antagonists map conceptually onto hyperarousal-dominant insomnia, whereas melatonergic therapies align more closely with circadian-disruption phenotypes. This target–phenotype correspondence may improve candidate prioritization, reduce therapeutic imprecision, and enhance signal detection in future clinical trials (Ren et al., 2025).

### 6.3 Repurposing opportunities in perioperative symptom-cluster management

In addition to novel or emerging hypnotics, repurposing of existing perioperative and symptom-cluster therapies represents an important and clinically practical direction for therapeutic development in PSD after breast cancer surgery. This strategy is particularly relevant because PSD rarely occurs in isolation, but instead clusters with pain, fatigue, anxiety, inflammation, nocturnal discomfort, and medication-related effects (Lai et al., 2025).

Gabapentinoids illustrate this concept well. Although not primary hypnotics, gabapentin and pregabalin may improve sleep indirectly by reducing neuronal hyperexcitability, neuropathic symptoms, nocturnal discomfort, and pain-related arousal. Their translational value is therefore likely greatest in pain-dominant or mixed postoperative phenotypes rather than in isolated hyperarousal-related insomnia. In carefully selected patients, these agents may support both opioid-sparing pain control and improved sleep continuity, although their utility is limited by dizziness, somnolence, gait instability, renal dosing considerations, and increased respiratory risk when combined with opioids (Gold et al., 2025).

More broadly, multimodal analgesia should itself be considered a therapeutic development domain relevant to sleep outcomes (Chen J. et al., 2025). In perioperative breast cancer care, poor pain control worsens sleep continuity and hyperarousal, while sleep disruption may in turn intensify pain sensitivity and emotional distress. This bidirectional relationship supports inclusion of sleep-related endpoints in analgesic trials and pain-related endpoints in trials of postoperative insomnia interventions (Chen Y. et al., 2025).

Overall, repurposing strategies are attractive because they build on therapies already integrated into perioperative care. However, their future value will depend on development frameworks capable of distinguishing genuine sleep-modifying effects from nonspecific sedation, identifying phenotype-relevant subgroups, and incorporating multidimensional endpoints spanning sleep, pain, functional recovery, and safety (Varallo et al., 2022).

### 6.4 Priorities for phenotype-stratified clinical trials

The next phase of therapeutic development in PSD after breast cancer surgery should move toward phenotype-stratified clinical trials rather than broadly inclusive studies that group biologically distinct presentations under a single insomnia label. Patients with hyperarousal-dominant insomnia are unlikely to respond in the same way as those with pain-dominant, circadian-disrupted, or mixed symptom-cluster phenotypes, and failure to distinguish these groups may dilute treatment effects and obscure clinically relevant safety signals (Wang and Sofer, 2025).

A practical framework for early trial enrichment could include at least three clinically actionable phenotypes: hyperarousal-dominant insomnia, circadian-disruption phenotypes, and pain-dominant or mixed symptom-cluster phenotypes (Zhu and Xue, 2025). Hyperarousal-dominant groups may be most suitable for DORAs or selected short-course hypnotics; circadian-disruption phenotypes for melatonin receptor agonists or combined chronobiologic interventions; and pain-dominant or mixed presentations for gabapentinoids, multimodal analgesic pathways, or integrated symptom-cluster strategies.

These phenotypes need not initially rely on complex biomarker panels, as clinically accessible variables such as sleep timing irregularity, pain burden, opioid exposure, nocturnal awakenings, and concurrent affective distress may already provide a practical basis for stratification (Lee et al., 2025).

Biomarkers should increasingly be incorporated as tools for mechanistic refinement and response assessment. Among currently available candidates, IL-6, CRP, cortisol rhythm measures, melatonergic timing markers, and selected autonomic indicators appear particularly relevant (Skubic et al., 2025).

Importantly, safety outcomes should be evaluated as rigorously as efficacy outcomes, particularly respiratory events, oversedation, falls, next-day cognitive impairment, and cumulative sedative exposure (Guo et al., 2025). Future trials should also incorporate multidimensional endpoints beyond sleep alone, including pain interference, fatigue, quality of life, daytime functioning, opioid consumption, and recovery-related outcomes (Su et al., 2025).

Overall, the next phase of therapeutic development should move toward a selective and development-oriented framework in which candidate agents are prioritized according to dominant biological drivers, tested in phenotype-enriched populations, and evaluated using integrated efficacy, safety, and mechanistic endpoints. Such a framework is more likely to generate clinically actionable signals than broadly inclusive insomnia trials that overlook the biological and pharmacologic heterogeneity of postoperative breast cancer PSD. Key pharmacologic classes, phenotype-guided positioning, evidence gaps, and development priorities are summarized in Table 4.

**TABLE 4:** Development landscape and phenotype-guided therapeutic positioning of pharmacologic strategies for postoperative sleep disturbance (PSD) after breast cancer surgery.

Drug/Class	Core target/Mechanism	Best-fit PSD phenotype	Direct evidence in postoperative breast cancer PSD	Supportive evidence outside breast cancer	Major safety concerns	Endocrine therapy relevance	Development priority/Key knowledge gap
Dual orexin receptor antagonists (DORAs)	OX1/OX2 receptor blockade; suppression of wake-promoting orexin signaling	Hyperarousal-dominant insomnia with difficulty initiating or maintaining sleep	Absent or very limited	Strong evidence from primary insomnia populations; limited perioperative oncology-specific data	Daytime somnolence, additive CNS depression, uncertain safety with concurrent opioid exposure	No established tamoxifen-specific concern	High — highly promising wake-targeted strategy; requires phenotype-enriched trials with recovery, daytime functioning, and safety endpoints
Melatonin/melatonin receptor agonists	MT1/MT2 receptor activation; circadian phase realignment	Circadian-disruption phenotype, delayed sleep phase, irregular sleep–wake timing	Limited	Supportive evidence from breast cancer sleep studies and broader perioperative circadian settings	Variable response depending on timing, environmental light exposure, and circadian instability; limited long-term postoperative data	No major tamoxifen-specific concern established	High — requires timing-sensitive, phenotype-guided trials using objective sleep and circadian endpoints
Gabapentinoids	Binding to the α2δ subunit of voltage-gated calcium channels; reduced excitatory neurotransmission	Pain-dominant or mixed symptom-cluster phenotype with nocturnal discomfort or neuropathic symptoms	Very limited	Supportive evidence from neuropathic pain and perioperative pain-related sleep disturbance in other populations	Dizziness, gait instability, oversedation, respiratory depression with opioids; renal dose adjustment may be required	No major endocrine-specific interaction, but cumulative sedative burden is important	Selective priority — strong repurposing candidate for pain-related arousal; requires phenotype-guided trials with opioid-sparing and sleep-related endpoints
Multimodal analgesic strategies	Opioid-sparing analgesic optimization targeting nociceptive, inflammatory, and neuropathic contributors	Pain-dominant or mixed symptom-cluster phenotype	Indirect	Strong perioperative rationale across multiple surgical settings; sleep outcomes often secondary	Regimen-dependent adverse effects; opioid co-exposure and cumulative sedation remain central concerns	Usually indirect relevance	High — major pragmatic development priority; requires perioperative trials with standardized sleep and symptom-cluster outcomes
Antidepressant-based sleep strategies	Serotonergic and noradrenergic modulation; in some agents, 5-HT2A antagonism and sedative effects	Insomnia with coexisting affective distress or mixed insomnia–distress presentations	Very limited	Indirect supportive evidence from oncology and chronic insomnia populations	Sedation, dizziness, orthostasis, next-day cognitive impairment, drug–drug interactions	High relevance — strong CYP2D6 inhibitors (e.g., paroxetine, fluoxetine) may reduce tamoxifen efficacy	Selective — requires clearer phenotype positioning based on psychiatric symptom burden and endocrine treatment context
Benzodiazepines/Z-drugs	Positive allosteric modulation of GABA-A receptors	Acute hyperarousal-dominant insomnia requiring short-term rescue treatment	Very limited	Broad insomnia and perioperative clinical experience	Dependence, falls, cognitive impairment, respiratory risk with opioids, residual sedation	No major endocrine-specific concern; polypharmacy remains important	Moderate — established short-term rescue role, but limited future development rationale beyond safer perioperative positioning
Cannabinoid-based agents	CB1/CB2 receptor modulation affecting pain, arousal, and inflammatory signaling	Selected mixed symptom-cluster phenotypes with pain or distress	Very limited	Variable evidence from non-breast-cancer oncology and chronic symptom settings	Sedation, neurocognitive adverse effects, product variability, uncertain interaction profile	Potential pharmacokinetic and pharmacodynamic interaction concerns	Exploratory — requires higher-quality efficacy, safety, and interaction studies
Atypical antipsychotics	Multi-receptor antagonism involving 5-HT2A, H1, and D2 receptors	Refractory insomnia with strong affective or treatment-related indications	Minimal	Limited indirect evidence	Metabolic adverse effects, QT prolongation, extrapyramidal symptoms, excessive sedation	No major tamoxifen-specific advantage; substantial polypharmacy concerns	Low — limited rationale for development as primary insomnia therapy
Sedating antihistamines/OTC sedatives	Central H1 receptor blockade with anticholinergic effects in some agents	Transient short-term insomnia without strong phenotype specificity	Minimal	Common real-world use but weak formal development rationale	Anticholinergic burden, cognitive impairment, residual daytime sedation, tolerance	OTC duplication may complicate supportive care and medication review	Low — limited rationale for formal therapeutic development

This table summarizes the current pharmacologic development landscape for postoperative sleep disturbance after breast cancer surgery, emphasizing phenotype-guided therapeutic positioning, available evidence, major safety considerations, endocrine therapy relevance, and key knowledge gaps for future research.

Abbreviations: PSD, postoperative sleep disturbance; DORA, dual orexin receptor antagonist; GABA-A, gamma-aminobutyric acid type A; MT1/MT2, melatonin receptor 1/2; OX1/OX2, orexin receptor 1/2; CNS, central nervous system; 5-HT2A, 5-hydroxytryptamine receptor 2A; H1, histamine-1, receptor; CB1/CB2, cannabinoid receptor 1/2; OTC, over-the-counter; CYP2D6, cytochrome P450 2D6.

## 7 Limitations and evidence gaps

Despite the growing mechanistic and therapeutic literature, several limitations continue to constrain translation to PSD after breast cancer surgery. Definitions of sleep disturbance remain heterogeneous across studies, and objective assessments such as actigraphy or polysomnography are used inconsistently, limiting comparability across cohorts and reducing precision in phenotype characterization. In addition, much of the available evidence is extrapolated from broader breast cancer survivorship or mixed oncology populations rather than from clearly defined postoperative breast cancer cohorts.

Many studies are further limited by small sample sizes, short follow-up duration, heterogeneous interventions, and variable control conditions, particularly in non-pharmacologic and integrative research. Importantly, safety reporting remains incomplete across the literature, especially with respect to sedative polypharmacy, opioid co-exposure, cognitive adverse effects, respiratory risk, and clinically relevant drug-drug or herb-drug interactions in the context of endocrine therapy and multimodal postoperative care.

These limitations underscore the need for adequately powered, phenotype-stratified clinical trials using standardized sleep outcomes, objective monitoring where feasible, and integrated assessment of efficacy, safety, biomarkers, and recovery-related endpoints.

## 8 Conclusion and future perspectives

PSD after breast cancer surgery is a clinically important and biologically heterogeneous condition that substantially affects postoperative recovery, symptom burden, and quality of life. Current evidence supports the rationale for a phenotype-guided management framework in which pharmacologic management is used selectively and with careful safety oversight, while behavioral, circadian-oriented, and integrative strategies are layered to improve longer-term outcomes and reduce unnecessary sedative exposure; however, this framework should be interpreted as hypothesis-generating until prospectively validated in postoperative breast cancer cohorts.

Future progress in breast cancer care will depend on more precise characterization of clinically relevant postoperative sleep phenotypes and the development of individualized therapeutic strategies that account for pain burden, circadian disruption, affective symptoms, endocrine therapy context, and cumulative medication-related risk. Particular priority should be given to phenotype-enriched clinical trials evaluating target-aligned pharmacologic interventions, including orexin receptor antagonists, melatonergic agents, and repurposed perioperative therapies within symptom-cluster management frameworks.

Further studies should focus on clearly defined postoperative breast cancer populations, standardized sleep endpoints, objective monitoring where feasible, and rigorous safety assessment, particularly with respect to opioid exposure, cumulative sedative burden, next-day cognitive impairment, and functional recovery. Over time, clinically accessible markers such as IL-6, CRP, cortisol rhythm, and circadian timing indicators may contribute to risk stratification, therapeutic positioning, and response monitoring.

Overall, progress in this field will require moving beyond nonspecific symptomatic sedation toward a more precise, mechanism-informed, phenotype-guided, and safety-conscious pharmacologic management model for women undergoing breast cancer surgery.
